# The imbalance of Th17/Treg cells exists in asymptomatic hyperuricemia (the early stage of gout)

**DOI:** 10.1007/s10067-026-07939-w

**Published:** 2026-01-16

**Authors:** Li-qing Zhang, Li-jun Zhao, An-lin Qin, Qian-ying Wen, Chong Gao, Xiao-feng Li

**Affiliations:** 1 Department of Rheumatology, The Second Hospital of Shanxi Medical University, Taiyuan, 030001 China; 2 Department of Rheumatology, Fenyang Hospital in Shanxi Province, Fenyang, China; 3 Shanxi Medical University, Taiyuan, China; 4 Department of Pathology, Brigham and Women’s Hospital, Harvard Medical School, Boston, USA

**Keywords:** Asymptomatic hyperuricemia, CD4 + T cells, Gout, Th17

## Abstract

**Objectives:**

Given that hyperuricemia is a metabolic condition with a prolonged asymptomatic period and strong associations with gout and various metabolic disorders, we investigated the role of lymphocyte subsets in asymptomatic hyperuricemia (aHUA) and explored their potential implications for immune regulation.

**Method:**

The study enrolled 59 male patients with aHUA, 29 with acute gout (AG), and 28 healthy male controls (HCs). Laboratory data, including blood cell counts, inflammatory markers, blood lipids, liver and renal function, and the percentage and absolute counts of lymphocytes and CD4 + T cell subpopulations in peripheral blood, were collected. We used flow cytometry to assess the peripheral blood lymphocyte subsets in these participants.

**Results:**

There were significant differences in GGT levels among all three groups, with the aHUA group showing the lowest value (AG vs. aHUA vs. HC, 69.00 vs. 23.00 vs. 35.50; *P* < 0.001). The lymphocyte subset data revealed a significant increase in the counts of helper T2 (Th2) (AG vs. aHUA vs. HC, 10.73 vs. 10.63 vs. 5.78; *P* < 0.001), Th17 (AG vs. aHUA vs. HC, 16.86 vs. 10.23 vs. 7.78; *P* < 0.001), and T suppressor (Ts) cells (AG vs. aHUA vs. HC, 669.32 vs. 655.00 vs. 488.84; *P* = 0.023), as well as the Th17/Treg ratio (AG vs. aHUA vs. HC, 0.44 vs. 0.37 vs. 0.26; *P* < 0.001) in both aHUA and AG groups. Furthermore, the increase in Th17 cells and the Th17/Treg ratio was more pronounced in the AG group. Total T cell levels were higher in both the aHUA and AG groups than in HCs, with the aHUA group showing the highest levels (statistically significant versus HCs) (AG vs. aHUA vs. HC, 1517.00 vs. 1620.00 vs. 1316.10; *P* < 0.001). Furthermore, the univariate regression analysis suggests that GGT [OR (95% CI) = 1.132 (1.069, 1.198), *P* < 0.001], Th17 [OR (95% CI) = 1.228 (1.104, 1.366), *P* < 0.001], and the Th17/Treg ratio [OR (95% CI) = 18.900 (1.892, 188.833), *P* = 0.012] are positively associated with acute gout flare. Multivariate regression analysis indicated that GGT [OR (95% CI) = 1.113 (1.049, 1.181), *P* < 0.001] and Th17 [OR (95% CI) = 1.235 (1.033, 1.476), *P* = 0.020] are positively correlated with acute gout flare.

**Conclusions:**

Our study highlights significant alterations in lymphocyte subsets in aHUA, emphasizing their potential role in the immune response and providing insights for future therapeutic strategies.

**Table:** 

**Key Points** •* Th17 elevation and Th17/Treg imbalance in asymptomatic hyperuricemia (aHUA) suggest early immune dysregulation.* • *Th17 cell levels and GGT are positively associated with acute gout flares, serving as biomarkers of disease activity.* • *Both aHUA and gout patients show immune activation, while there were immunological differences across groups.*

## Introduction

Hyperuricemia (HUA) is defined by elevated serum uric acid levels, typically exceeding 6 mg/dL in women and 7 mg/dL in men [1]. With changes in modern lifestyles, the prevalence of hyperuricemia has gradually increased [2]. Approximately two-thirds or more of such individuals remain asymptomatic. Despite this, persistent hyperuricemia is considered an independent risk factor for various health complications, including cardiovascular diseases, kidney disorders, and metabolic syndrome [3, 4], all of which contribute to significant healthcare costs and a reduced quality of life [5]. Current therapeutic approaches, such as lifestyle changes and urate-lowering treatments, face challenges related to patient compliance and potential side effects [6]. Therefore, it is vital to investigate the underlying mechanisms of asymptomatic hyperuricemia (aHUA).

The prevalence of asymptomatic hyperuricemia (aHUA) has garnered significant attention due to its association with various health complications, particularly gout [1, 7]. Gout is characterized by recurrent inflammatory arthritis. This condition results from the deposition of monosodium urate (MSU) crystals in tissues, which occurs due to elevated serum uric acid levels. Uric acid exists mainly as soluble urate (sUA) and monosodium urate (MSU) crystals. The development and complications of hyperuricemia (HUA) result from complex interactions among these forms and the innate immune system [8]. AHUA often precedes gout; however, many individuals remain unaware of the risks posed by elevated uric acid levels until symptoms develop. Understanding the immunopathological implications of aHUA is critical, as it may lead to chronic conditions if left untreated [9–11].

Recent studies have emphasized the critical role of immune responses, particularly T lymphocyte subsets, in the pathogenesis of hyperuricemia and gout, thereby providing important insights into the underlying immune mechanisms in affected patients [12–15]. Zi et al. reported that during the early stages of gout, the proportion of Th17 cells and the Th17/Treg ratio were significantly elevated. This finding indicates that Th17-mediated immune responses play a critical role in gout-related inflammation. Th17 cells contribute to the pathogenesis of gout by secreting interleukin-17 (IL-17), which promotes neutrophil recruitment and enhances the release of inflammatory cytokines, thereby exacerbating joint inflammation [14]. Similarly, Zhao et al. compared CD4^+^ T-lymphocyte subsets in the acute and chronic stages of gout and found a marked increase in Th17 cells—particularly in the chronic stage—accompanied by a decrease in Treg cells, resulting in an imbalance of the Th17/Treg ratio and further amplification of the inflammatory response. In addition, a significant elevation of Th1 cells in peripheral blood was observed, suggesting that Th1-mediated immune responses also play a major role in gout-associated inflammation [15]. In addition, the changes of metabolic markers like liver enzyme during this process have also attracted attention. A cross-sectional study has shown that BMI is associated with hyperuricemia, and abnormal liver enzymes may play a mediating role [16]. Furthermore, adults with hyperuricemia and gout are most likely to develop liver dysfunctions and suffer associated morbidities [17]. However, a systematic analysis of the specific immune mechanisms and their association with underlying aHUA remains lacking. Therefore, an in-depth exploration of these mechanisms, particularly the changes in lymphocyte subsets and their association with metabolic markers, holds significant clinical implications.

This study employs a cross-sectional design to assess the specific lymphocyte subset profiles of male patients diagnosed with aHUA, individuals undergoing acute gout attacks (AG), and healthy controls (HCs). Additionally, since HUA is frequently associated with chronic comorbidities such as hypertension, dyslipidemia, and diabetes [18, 19], further investigation into these associations is warranted. This study also examines the correlation between T lymphocyte subsets and blood biochemical markers associated with aHUA. Through these analyses, we aimed to elucidate potential immune dysregulation associated with aHUA, thereby paving the way for targeted interventions in at-risk populations.

## Materials and methods

### Participants

We selected aHUA and AG patients admitted to Fenyang Hospital in Shanxi Province (Lvliang, China) from July 2022 to July 2023.

The inclusion criteria were as follows:

- Aged between 25 and 60 years.
- For the aHUA group: patients who met the diagnostic criteria of the Chinese Guidelines for the Diagnosis and Treatment of Hyperuricemia and Gout (2019), defined as serum uric acid (SUA) levels exceeding 420 μmol/L on two separate days, without a history of gout attacks.
- For the AG group: patients who fulfilled the 2015 American College of Rheumatology/European League Against Rheumatism (ACR/EULAR) gout classification criteria, experiencing their first episode of acute gouty arthritis, characterized by marked redness, swelling, heat, and pain in peripheral joints with laboratory evidence of inflammation.
- For the AG group: blood samples were strictly collected within 24 to 48 h of symptom onset.
- Patients with complete demographic information, physical examination findings, and laboratory test data.
- Patients who voluntarily participated in the study and provided written informed consent.
- Patients with comorbidities such as diabetes, dyslipidemia, hypertension, or hyperuricemia who had not received any medication other than basic antihypertensive or antidiabetic treatment, and had not used any relevant drugs such as colchicine, non-steroidal anti-inflammatory drugs (NSAIDs), or corticosteroids within 2 months prior to enrollment.

The exclusion criteria were as follows:

- Patients with cardiovascular or cerebrovascular diseases, acute nephritis, chronic kidney disease (CKD) stage 3 or higher, infectious or non-infectious liver disease, inflammatory bowel disease, irritable bowel syndrome, severe dyslipidemia, uncontrolled hypertension, or diabetes with complications.
- Patients with other autoimmune or autoinflammatory diseases, allergic diseases, immunodeficiency disorders, or severe infections.
- Patients who had used urate-lowering drugs, immunosuppressants, corticosteroids, probiotics or similar microecological preparations, or antibiotics within 1 month before enrollment.

**Fig. 1:**
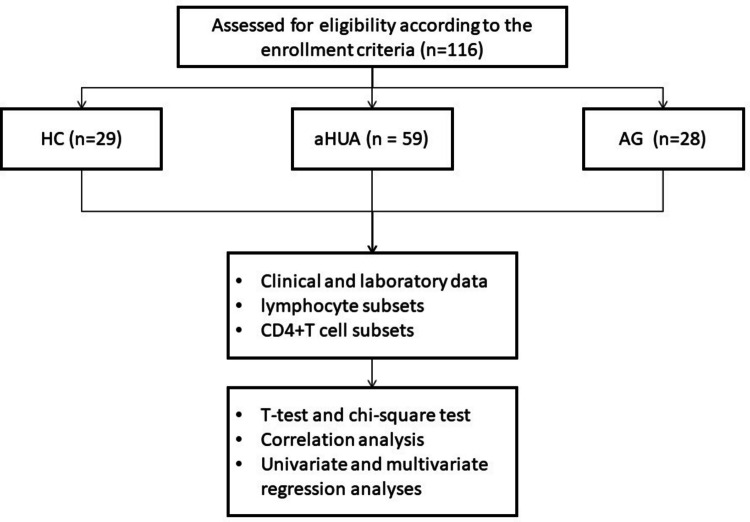
Study flowchart

In a small number of cases where patients required immediate pain relief on the day of consultation but were awaiting fasting blood collection, topical flurbiprofen patches were applied locally. All aHUA and gout patients except one were male, so we skipped the 1 female gout patient. Finally, a total of 59 male aHUA patients and 28 male AG patients were recruited for this study. Additionally, 29 healthy individuals, matched for age with the aHUA and AG patients, were also enrolled from Fenyang Hospital as HCs. All participants met the inclusion and exclusion criteria and provided the informed consent, and the study was approved by the Ethics Committee of Fenyang Hospital, Shanxi (Approval Number: 2022045). The study flowchart is illustrated in Fig. 1.

### Clinical data

Demographic and disease features, including age, BMI, and comorbidities (hypertension and diabetes), were recorded. Laboratory data, including erythrocyte sedimentation rate (ESR), alanine transaminase (ALT), aspartate aminotransferase (AST), γ-glutamyl transpeptidase (GGT), serum uric acid (SUA), serum creatinine (Cr), total cholesterol (CHO), triglycerides (TG), and fasting blood glucose (FBG), as well as percentages and absolute counts of lymphocytes and CD4 + T cell subpopulations in the blood, were also collected.

### Analysis of circulating lymphocyte and CD4 + T cell subpopulations

The flow cytometer was set up using BD FACSDiva™ CS&T Research Beads, and quality control was performed with BD Multi-Check™ Control. Flow cytometry compensation was conducted using BD FACS™ 7-color Setup Beads.

#### Determination of absolute counts of lymphocyte subsets

Peripheral blood lymphocyte subsets include total T lymphocytes, total B lymphocytes, natural killer (NK) cells, CD4 + T cells, and CD8 + T cells.

**Fig. 2:**
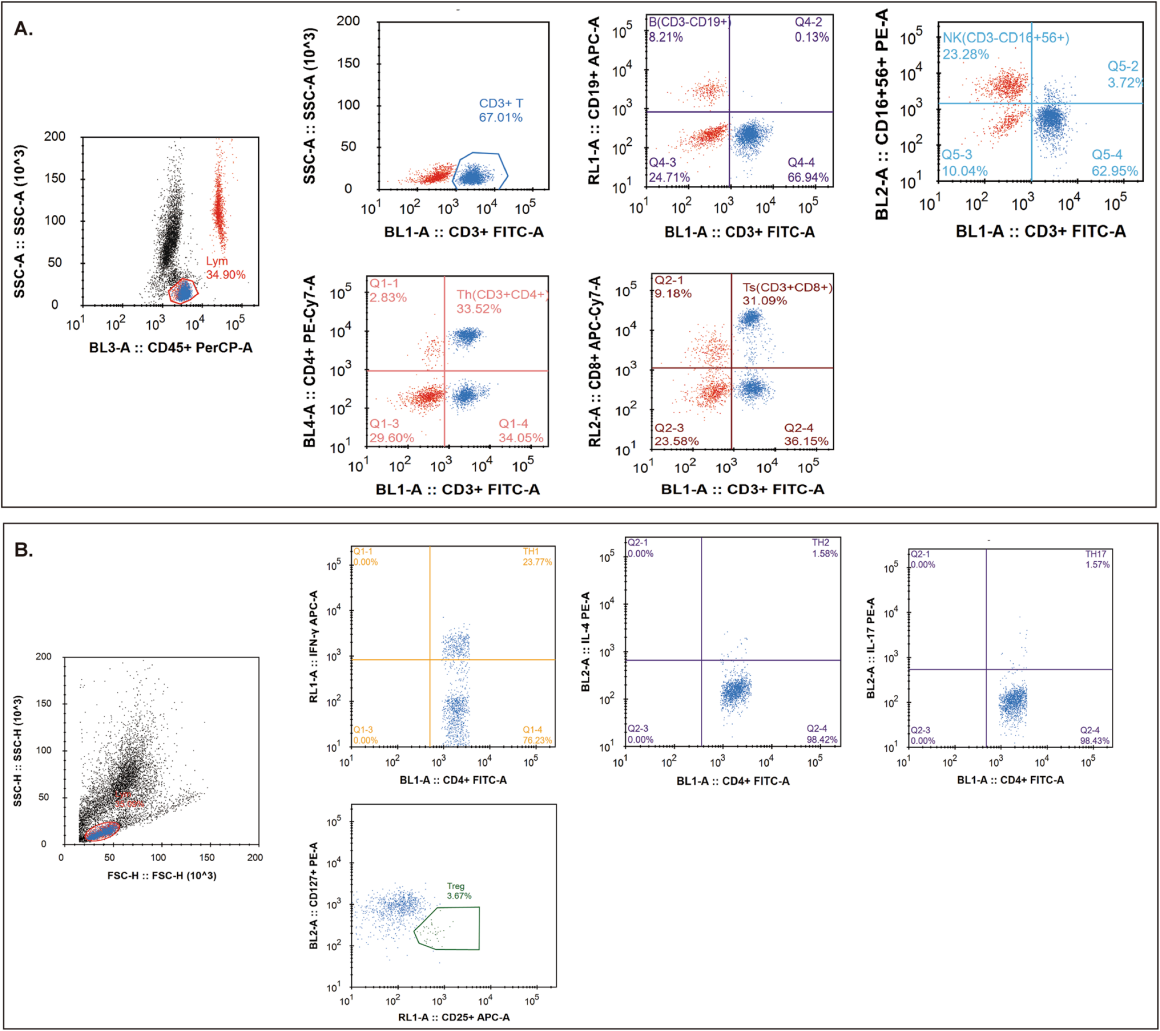
Representative diagram of the gating for flow cytometric analysis of lymphocyte and CD4 + T subsets. **A** Flow cytometry analysis of peripheral lymphocytes. T cells: CD45 + CD3 + CD19 −, B cells: CD45 + CD3 − CD19 +, NK cells: CD45 + CD3 − CD16 + CD56 +, CD4 + T cells: CD45 + CD3 + CD4 +, CD8 + T cells: CD45 + CD3 + CD8 +. **B** Flow cytometry analysis of CD4 + T cell subsets. Th1 cells: CD4 + IFN-γ +, Th2 cells: CD4 + IL-4 +, Th17 cells: CD4 + IL-17 +, Treg cells: CD4 + CD25 + CD127^low^

The specific method was as follows: two flow tubes, labeled A and B, each containing a known of fluorescent microspheres, were prepared. Then, 50 μL of peripheral blood was added using the reverse sampling method. Twenty microliters of the anti-CD3 FITC/CD8 PE/CD45 PerCP/CD4 APC antibody and 20 μL of the CD3 FITC/CD16 + 56 PE/CD45 PerCP/CD19 APC antibody were added to tubes A and B, respectively. The mixture was thoroughly mixed and incubated at room temperature in the dark for 20 min. Subsequently, 450 μL of XFACS hemolysin was added and incubated under the same conditions for 15 min. Each sample recorded a total of 15,000 events (Fig. 2). The principle is as follows: a known quantity of approximately 50,000 microspheres (the exact number indicated on the box) was placed in advance in the absolute counting tube, and 50 μL of whole blood was added. This ensured a fixed ratio of blood to microspheres in the sample, allowing calculation of the number of lymphocytes in 50 μL of whole blood, and subsequently the number of lymphocytes per microliter. Absolute counts (cells/μL) were calculated as:

$$Cell\left(\frac{cell}{{\mu \mathrm{L}}}\right)=\frac{E{}_{cell}}{{E}_{beads}}\times \frac{{N}_{beads}}{{V}_{sample}}$$

where $E{}_{cell}$ and ${E}_{bead}$ are the acquired events for the target cell population and counting beads by FCM, ${N}_{bead}$ is the manufacturer-specified bead count per tube, and ${V}_{sample}$ is the sample volume added to the tube (50 ${\mu\mathrm{L}}$ in our research).

#### Determination of absolute counts of CD4 + T-cell subsets

Peripheral blood CD4 + T-cell subsets include Th1 cells, Th2 cells, Th17 cells, and Treg cells. The specific methods were as follows: 10 μL of phorbol myristate acetate working solution, 10 μL of ionomycin working solution, and 1 μL of GolgiStop™ were added to 80 μL of peripheral blood samples and incubated at 37 °C in an incubator containing 5% CO2 for 5 h. Samples were stained with anti-CD4-FITC at room temperature, protected from light, for 30 min. One milliliter of freshly prepared fixing and permeabilizing solution was added and incubated at 4 °C for 30 min, protected from light. Th1 cells were detected using anti-CD4-FITC and anti-IFN-γ-APC antibodies; Th2 cells with anti-CD4-FITC and anti-IL-4-PE antibodies; and Th17 cells with anti-IL-17-PE antibodies. Treg cells were incubated with the CD4-FITC antibody and CD25-APC antibody in 80 μL of blood samples at room temperature for 30 min away from light, then 1 mL of freshly prepared fixed/permeable mixture solution was added, and the cells were incubated with anti-CD127-PE under the same conditions. The CD127^low/−^ phenotype was utilized for isolating Tregs because this surface-marker-based approach ensures high cell viability and experimental reproducibility [20]. Finally, the cells were washed twice with phosphate-buffered saline (PBS). Isotype controls were established using the same procedure. All immunofluorescence antibodies were purchased from BD Biosciences. All stained cells were analyzed using flow cytometry (FACSCanto II; BD Biosciences, Franklin Lakes, NJ, USA). The data were analyzed using *FlowJo* V7.6.1 (Tree Star Inc., Ashland, OR, USA). The absolute number of cells in each subpopulation was calculated by multiplying the percentage of counted cells by the absolute number of CD4 + T lymphocytes. The markers of each CD4 + T-cell subpopulation are shown in Fig. 2.

### Statistical analysis

Statistical analysis was performed using IBM SPSS Statistics, version 27.0. Continuous variables were summarized as mean ± standard deviation (mean ± SD) if normally distributed and as median (interquartile range, IQR) otherwise. Categorical variables were expressed as counts and percentages. For two-group comparisons, if variables were normally distributed with homogeneous variances (assessed by Shapiro–Wilk and Levene’s tests), the independent-samples *t*-test was used. Otherwise, the Mann–Whitney *U* test was applied. For three-group comparisons, normally distributed data were analyzed by one-way ANOVA (with Tukey’s HSD for post hoc pairwise comparisons). Non-normally distributed or variance-unequal data were analyzed by the Kruskal–Wallis *H* test. When the omnibus test was significant, Mann–Whitney *U* pairwise tests with Bonferroni-adjusted *P* values were performed. For categorical data, group differences were tested using the chi-square test; when any expected cell count was < 5, Fisher’s exact test *P* value was reported. Correlations were assessed using Pearson’s *r* (Pearson correlation coefficient) for normally distributed variables and Spearman’s *ρ* (Spearman’s rank correlation coefficient) otherwise. To evaluate factors associated with gout flare (binary outcome), univariable and multivariable logistic regression analyses were performed. Variables with clinical relevance and/or *P* < 0.10 in univariable analyses entered the multivariable model, with multicollinearity checked by variance inflation factors (VIFs). All tests were two-tailed with an alpha level (*α*) = 0.05.

## Results

### Comparison of clinical data among three groups

As shown in Table 1, there were no significant differences in age among the three groups (*P* = 0.318). BMI was significantly higher in the AG group compared to the HCs (AG vs. HC, 27.1 vs. 25.10; *P* = 0.045). Liver function markers, such as ALT, did not differ significantly across groups (AG vs. aHUA vs. HC, 30.00 vs. 25.00 vs. 28.00; *P* = 0.458); however, AST was significantly elevated in the AG group compared to HCs (AG vs. aHUA vs. HC, 22.00 vs. 25.00 vs. 19.50; *P* = 0.011). Additionally, GGT levels showed significant variation among all three groups (AG vs. aHUA vs. HC, 69.00 vs. 23.00 vs. 35.50; *P* < 0.001), with the AG group exhibiting the highest median value. SUA levels were significantly higher in both the aHUA and AG groups than in the HCs (AG vs. aHUA vs. HC, 475.00 vs. 490.00 vs. 298.50; *P* < 0.001), as were creatinine (Cr) levels (AG vs. aHUA vs. HC, 80.00 vs. 77.00 vs. 55.00; *P* < 0.001). TG levels were also significantly elevated in both the aHUA and AG groups (AG vs. aHUA vs. HC, 1.72 vs. 1.75 vs. 1.20; *P* < 0.001), while total CHO levels showed no statistically significant differences (AG vs. aHUA vs. HC, 4.85 vs. 4.62 vs. 4.41; *P* = 0.059). Regarding comorbidities, there is no difference in the prevalence of hypertension (*P* = 0.130) and diabetes (*P* = 0.129) among the three groups.

**Table 1:** Baseline characteristics of all subjects

	aHUA (*n* = 59)	AG (*n* = 29)	HCs (*n* = 28)	*H*	*P*
Age (years)	40.00 (34.00, 44.00)	38.00 (30.00, 54.00)	41.50 (37.00, 55.75)	2.290	0.318
BMI (kg/m^2^)	25.90 (25.10, 27.67)	27.21 (25.40, 28.67)^c^	25.10 (22.73, 28.80)	6.223	0.045*
ALT (U/L)	25.00 (20.00, 35.00)	30.00 (19.60, 45.35)	28.00 (20.25, 37.50)	1.560	0.458
AST (U/L)	25.00 (19.00, 30.00)	22.00 (18.70, 29.80)^c^	19.50 (16.00, 23.75)	9.023	0.011*
GGT (U/L)	23.00 (17.00, 30.00)^a^	69.00 (43.00, 118.50)^bc^	35.50 (22.25, 40.00)	47.065	< 0.001*
SUA (μmol/L)	490.00 (469.00, 554.00)^a^	475.00 (361.00, 562.50)^c^	298.50 (267.25, 348.75)	54.439	< 0.001*
Cr (μmol/L)	77.00 (57.00, 85.00)^a^	80.00 (70.00, 123.50)^c^	55.00 (53.00, 66.00)	34.435	< 0.001*
CHO (mmol/L)	4.62 (3.92, 5.50)	4.85 (4.32, 5.74)	4.41 (3.94, 5.02)	5.675	0.059
TG (mmol/L)	1.75 (1.19, 2.44)^a^	1.72 (1.27, 2.20)^c^	1.20 (0.84, 1.61)	17.197	< 0.001*
Hypertension					
Yes (*n*, %)	8 (13.56%)	3 (10.34%)	0 (0%)	—	0.130
No (*n*, %)	51 (86.44%)	26 (89.66%)	0 (0%)		
Diabetes					
Yes (*n*, %)	5 (8.47%)	4 (13.79%)	0 (0%)	—	0.144
No (*n*, %)	54 (91.53%)	25 (86.21%)	0 (0%)		

*aHUA* asymptomatic hyperuricemia, *AG* acute gout attack, *HCs* healthy controls, *BMI* body mass index, *ALT* alanine aminotransferase, *AST* aspartate aminotransferase, *GGT* gamma-glutamyl transferase, *SUA* uric acid, *Cr* creatinine, *CHO*, cholesterol, *TG* triglyceride; continuous variables are presented as median (interquartile range, IQR) and the rest statistical values are *H*; when the omnibus test was significant, Mann–Whitney *U* pairwise tests with Bonferroni correction were performed. Categorical variables are shown as *n* (%); when any expected cell count < 5, Fisher’s exact test was used (only the *P* value is reported; the test-statistic column is shown as “—”). Superscripts indicate significant pairwise differences: ^a^aHUA vs. HCs; ^b^aHUA vs. AG; ^c^AG vs. HCs. *P < 0.05 was considered statistically significant

### Comparison of lymphocyte subsets among three groups

**Fig. 3:**
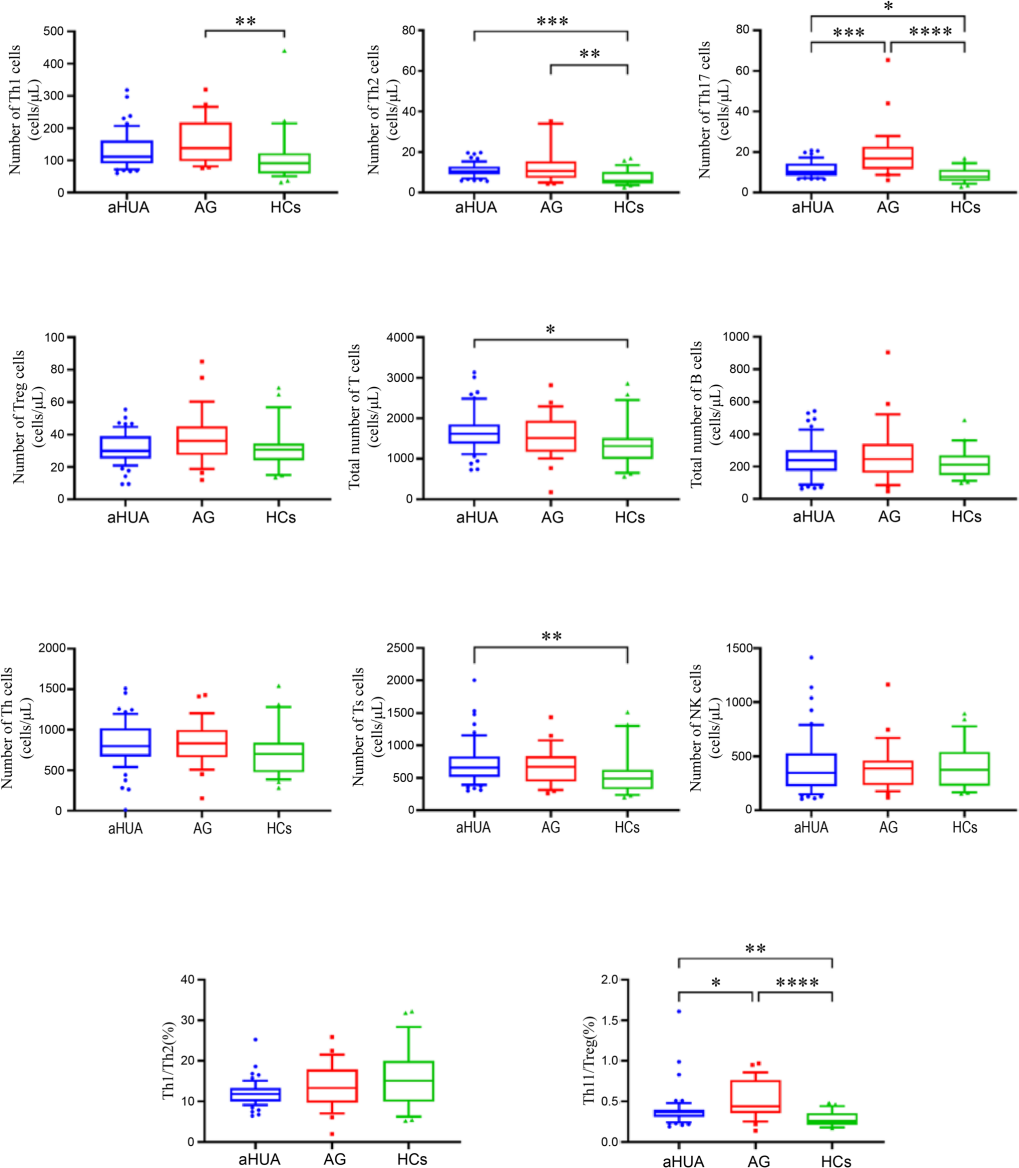
Box plots illustrate the distribution of various immune cell subsets across the three groups: aHUA (blue), AG (red), and HC (green). The central line in each box denotes the median, the box edges represent the interquartile range. Significance levels are indicated as follows: *P < 0.05, **P < 0.01, ***P < 0.001, and ****P < 0.0001

The results show significant differences in the distribution of lymphocyte subsets listed in Table 2 between the groups (Table 2 and Fig. 3). Specifically, individuals with aHUA exhibited higher levels of total T cells, T suppressor (Ts) cells, Th2, Th17 cells, and the Th17/Treg (%) ratio compared to the HCs (*P* < 0.05). Additionally, the Ts, Th1, Th2, Th17 cells, and Th17/Treg (%) ratio were also more abundant in the AG group, with statistically significant differences compared to the HCs (*P* < 0.05). Moreover, the Th17 cells and Th17/Treg (%) ratio in the aHUA group were lower than those in the AG group.

**Table 2:** Differences in levels of lymphocyte subsets with aHUA, AG, and HCs

Group	aHUA (*n* = 59)	AG (*n* = 29)	HCs (*n* = 28)	*F*/*H*	*P*
Total T (cells μl^−1^)	1620.00 (1374.00, 1852.00)^a^	1517.00 (1165.87, 1939.25)	1316.10 (990.08, 1518.08)	7.835	0.020*
Ts (cells μl^−1^)	655.00 (514.00, 831.00)^a^	669.32 (444.09, 837.37)^c^	488.84 (322.26, 626.83)	8.909	0.012*
Th (cells μl^−1^)	820.34 ± 279.10	830.57 ± 282.09	734.62 ± 303.19	1.045^d^	0.355
Th1 (cells μl^−1^)	111.60 (91.04, 161.97)	137.80 (97.54, 217.81)^c^	91.06 (59.48, 122.38)	10.343	0.006*
Th2 (cells μl^−1^)	10.63 (8.92, 12.83)^a^	10.73 (7.21, 15.40)^c^	5.78 (4.58, 10.22)	15.137	< 0.001*
Th17 (cells μl^−1^)	10.23 (8.47, 14.34)^a^	16.86 (11.59, 22.60)^bc^	7.78 (5.77, 11.38)	29.930	< 0.001*
Treg (cells μl^−1^)	29.79 (25.10, 39.02)	36.22 (27.40, 45.08)	30.59 (24.05, 34.60)	4.602	0.100
Th1/Th2 (%)	11.82 (9.97, 13.34)	13.31 (9.65, 17.89)	15.14 (9.95, 20.02)	5.970	0.051
Th17/Treg (%)	0.37 (0.31, 0.40)^a^	0.44 (0.36, 0.77)^bc^	0.26 (0.21, 0.36)	24.923	< 0.001*
Total B (cells μl^−1^)	239.00 (173.00, 300.00)	245.00 (160.59, 339.78)	210.29 (145.84, 267.99)	1.431	0.489
NK (cells μl^−1^)	347.00 (224.00, 526.00)	387.06 (231.64, 460.77)	375.50 (226.25, 539.79)	0.073	0.964

^a^aHUA vs. HCs, ^b^aHUA vs. AG, ^c^AG vs. HCs, ^d^indicates the value of *F,* *P < 0.05

### Correlation analysis between lymphocyte subsets and clinical indicators

A univariate regression analysis was conducted with acute gout flare status (AG group assigned a value of 1, representing an acute flare, and aHUA assigned a value of 0, representing no flare) as the dependent variable. Various biochemical indicators and lymphocyte subset parameters were used as independent variables. The univariate regression analysis suggests that GGT [OR (95% CI) = 1.132 (1.069, 1.198), *P* < 0.001], Th17 [OR (95% CI) = 1.228 (1.104, 1.366), *P* < 0.001], and the Th17/Treg ratio [OR (95% CI) = 18.900 (1.892, 188.833), *P* = 0.012] are positively associated with acute gout flare (Table 3). Further multivariate regression analysis indicated that GGT [OR (95% CI) = 1.113 (1.049, 1.181), *P* < 0.001] and Th17 [OR (95% CI) = 1.235 (1.033, 1.476), *P* = 0.020] are positively correlated with acute gout flare (Table 4).

**Table 3:** Univariate logistic regression

Variables	*B*	SE	Wald χ^2^	*P*	OR (95% CI)
GGT (U/L)	0.124	0.029	17.838	< 0.001*	1.132 (1.069, 1.198)
Th17 (cells μl^−1^)	0.206	0.054	14.338	< 0.001*	1.228 (1.104, 1.366)
Th17/Treg (%)	2.939	1.174	6.264	0.012*	18.900 (1.892, 188.833)

* *P* < 0.05

**Table 4:** Multivariate logistic regression

Variables	*B*	SE	Wald χ^2^	*P*	OR (95% CI)
GGT (U/L)	0.107	0.030	12.710	< 0.001*	1.113 (1.049, 1.181)
Th17 (cells μl^−1^)	0.211	0.091	5.389	0.020*	1.235 (1.033, 1.476)
Th17/Treg (%)	− 0.103	1.531	0.004	0.947	0.902 (0.045, 18.156)

* *P *< 0.05

## Discussion

This study aims to elucidate the role of lymphocyte subpopulations in individuals with aHUA, comparing their profiles with those of HCs and AG patients. Our results reveal significant alterations in lymphocyte subpopulations associated with aHUA, particularly in the context of Th17 cells and Th17/Treg ratio. These findings suggest that the immune system plays a crucial role in the pathophysiology of elevated uric acid levels, potentially influencing metabolic pathways of urate.

The observed decrease in Th17 cells in the aHUA group compared to the AG group indicates an association between these lymphocyte subpopulations and the inflammatory response seen in both aHUA and AG. This aligns with previous studies indicating that T cells, particularly Th17 cells, are key players in inflammatory diseases and may drive the immune responses that lead to crystal deposition and subsequent gout flare-ups [21]. These observations underscore the complex interplay between different immune subsets in autoimmune conditions and provide valuable insight into their potential relevance for future diagnostic research.

The increased total T cell count observed in the aHUA group suggests ongoing immune activation, which may not yet manifest clinically as acute inflammation, possibly due to early-stage compensatory mechanisms. This phenomenon could indicate the body’s compensatory mechanisms, which attempt to manage elevated uric acid levels through immune activation [12]. The relationship between total T cell levels and uric acid concentrations warrants further investigation, as it may provide insights into how T cell dynamics influence the progression from HUA to gout flares. Furthermore, the lack of significant differences in Treg levels across the groups, although higher levels were observed in AG, suggests a complex interaction between these regulatory cells and effector T cells that may influence the inflammatory response during gout attacks [14, 22]. Understanding these interactions may reveal new therapeutic strategies aimed at modulating immune responses in individuals predisposed to gout.

Previous research has established that the Th17/Treg balance plays a critical role in inflammatory processes and autoimmune diseases [23, 24]. Th17 cells promote inflammation, while Treg cells maintain immune homeostasis. In gout patients, Th17 cells are upregulated, Treg cells are downregulated, and the Th17/Treg ratio is positively correlated with joint inflammation [14, 15]. Targeted therapies that modulate the Th17/Treg balance have shown promise in treating gout [25]. Recent in vitro experiments have demonstrated that soluble urate can induce pro-inflammatory cytokines, such as IL-1β, IL-6, and TGF, even in the absence of MSU crystal deposition [26]. Additionally, patients with aHUA exhibit altered gut microbiota, characterized by an imbalanced Firmicutes/Bacteroidetes (F/B) ratio, which may disrupt the Th17/Treg equilibrium [27, 28]. This suggests a potential Th17/Treg imbalance in aHUA, which may contribute to chronic diseases such as diabetes and hypertension [3, 29–31]. This study found that both the aHUA and AG groups exhibited elevated levels of Th17 cells and Th17/Treg ratios, with the AG group showing higher levels than the aHUA group. The difference was statistically significant, suggesting that elevated levels of Th17 cells and Th17/Treg ratios are related to disease status.

Additionally, hyperuricemia is closely associated with abnormalities in lipid metabolism and liver function, and its prevalence is increasing among younger populations [9, 32–34]. This is also corroborated by our study data. The BMI of the AG group is significantly higher than that of the aHUA group, indicating a close correlation between BMI and the onset of gout attack. Uric acid can enhance lipid synthesis by triggering endoplasmic reticulum (ER) stress and activating transcription factors such as sterol regulatory element-binding protein 1 (SREBP-1) [35]. Elevated lipid levels may, in turn, promote inflammation and exacerbate oxidative stress [17]. Furthermore, uric acid can induce inflammation and oxidative stress, either directly or indirectly through lipids and glucose, ultimately leading to liver cell death and a reduction in functional hepatocytes [36]. These processes diminish the liver’s capacity to function properly, resulting in elevated liver enzyme levels like AST and GGT [16]. Interestingly, GGT levels are lower in the aHUA group, which may indicate a protective regulatory mechanism involving modulation of oxidative stress or inflammatory responses during the transition from aHUA to gout attack. This finding is noteworthy and warrants further investigation. Overall, managing body weight and regulating liver enzyme levels may play key roles in preventing and managing hyperuricemia.

The limitations of this study include a relatively small sample size and the inclusion of only male subjects, which may limit the generalizability of the findings. Additionally, the lack of longitudinal clinical validation limits understanding of the temporal dynamics of immune responses in relation to uric acid levels. Moreover, inflammatory cytokine levels were not assessed due to unavailability of such data from health check-up participants, restricting the depth of immune profile interpretation. Future studies should address this gap by incorporating cytokine measurements to provide a more comprehensive understanding of immune-inflammatory interactions. These limitations emphasize the need for larger, multi-center studies that incorporate clinical follow-up and experimental validation to strengthen the conclusions drawn from this research.

In conclusion, our research findings suggest that the immune system is involved in uric acid metabolism, and lymphocyte subsets play a significant role in the pathogenesis of aHUA and acute gout. Furthermore, our research also revealed that there are differential changes in lymphocyte subsets between aHUA and acute gout attacks. Future research should delve deeper into the molecular pathways and signaling mechanisms behind these immune alterations, as these insights could be key to developing new therapeutic strategies aimed at providing new directions for personalized treatment by targeting specific lymphocyte functions.

## Data Availability

Raw data used during the current study are available from the corresponding author, upon reasonable request.
